# The Impact of the Circadian Clock on Skin Physiology and Cancer Development

**DOI:** 10.3390/ijms22116112

**Published:** 2021-06-06

**Authors:** Janet E. Lubov, William Cvammen, Michael G. Kemp

**Affiliations:** Department of Pharmacology and Toxicology, Boonshoft School of Medicine, Wright State University, Fairborn, OH 45435, USA; lubov.2@wright.edu (J.E.L.); cvammen.2@wright.edu (W.C.)

**Keywords:** DNA repair, circadian clock, skin biology, skin cancer, genotoxicity, cell cycle, UV radiation

## Abstract

Skin cancers are growing in incidence worldwide and are primarily caused by exposures to ultraviolet (UV) wavelengths of sunlight. UV radiation induces the formation of photoproducts and other lesions in DNA that if not removed by DNA repair may lead to mutagenesis and carcinogenesis. Though the factors that cause skin carcinogenesis are reasonably well understood, studies over the past 10–15 years have linked the timing of UV exposure to DNA repair and skin carcinogenesis and implicate a role for the body’s circadian clock in UV response and disease risk. Here we review what is known about the skin circadian clock, how it affects various aspects of skin physiology, and the factors that affect circadian rhythms in the skin. Furthermore, the molecular understanding of the circadian clock has led to the development of small molecules that target clock proteins; thus, we discuss the potential use of such compounds for manipulating circadian clock-controlled processes in the skin to modulate responses to UV radiation and mitigate cancer risk.

## 1. Skin Cancers and Risk Factors

### 1.1. Melanoma and Non-Melanoma Skin Cancers

Skin cancers constitute the most common type of malignancy in the Caucasian population and include both melanomas and non-melanoma skin cancers (NMSCs), which are derived from melanocytes and keratinocytes, respectively [1,2]. Over the last 50 years, there has been an increase in the incidence of both melanomas and NMSCs, with a 0.6% annual increase in metastatic melanoma (MM). Although melanomas are less common than NMSCs, they are more dangerous because they spread hematogenously [1,3,4,5]. The prevalence of MM also increased in the last few years to a staggering 1.2 million cases in 2017 [2] with more than 100,000 new cases and nearly 7000 deaths in 2020 alone. There are four main types of melanomas: superficial, nodular, lentigo maligna, and acral lentiginous [3,4,5,6]. A MM begins in melanocytes in the basal layer of the epidermis and are most commonly found in regions of skin that are chronically exposed to the UV wavelengths of sunlight. UV radiation causes damage to DNA that can lead to mutations that activate oncogenes and inactivate tumor suppressor genes. However, melanomas can be found in other parts of the body. For example, in individuals with darker complexions, melanomas are often found in nail beds and on the soles of the feet [2,7].

In the United States, non-melanoma skin cancers (NMSCs) occur more frequently than melanomas and are the most common cancer, of which the basal-cell carcinoma (BCC) and squamous cell carcinoma (SCC) are the most common types. In Caucasians, NMSC has an 18-fold higher incidence than melanoma and has been attributed to living in specific geographic areas [1]. Although NMSCs have a lower mortality rate than MM, the estimated annual cost of treating an NMSC is 6 to 7 times higher [8]. In sun-exposed skin, NMSCs, like melanomas, contain UV signature mutations in different gene products that influence cell growth and proliferation [9].

### 1.2. Skin Cancer Risk Factors

As mentioned, melanomas and NMSCs are usually found in areas of sun-exposed skin, and thus UV exposure from sunlight or tanning beds is the most widely recognized cause of skin cancer. UV radiation is categorized as UV-A, -B, -C according to its electrophysical properties. UVC photons have the shortest wavelengths (100–280 nm) and highest energy and generally do not pose a risk to organisms in the biosphere as it is absorbed by atmospheric ozone. UVA photons have the longest (315–400 nm) but least energetic photons, and UVB falls in between with enough range and energy to maximally affect organisms in the biosphere. Because ambient sunlight predominantly consists of UVA and UVB photons, these wavelengths of light are particularly problematic for the epidermal cells of the skin. UVA and UVB radiation can damage DNA by generating reactive oxygen species via indirect photosensitizing reactions and by directly causing molecular rearrangements in DNA bases that form photoproducts such as cyclobutane pyrimidine dimers (CPDs). Although studies have shown that occupational and recreational exposure to UV radiation can increase the risk of cancer [10,11,12,13], most of these studies had limited controls for confounding variables that may affect skin cancer development.

**Figure 1:**
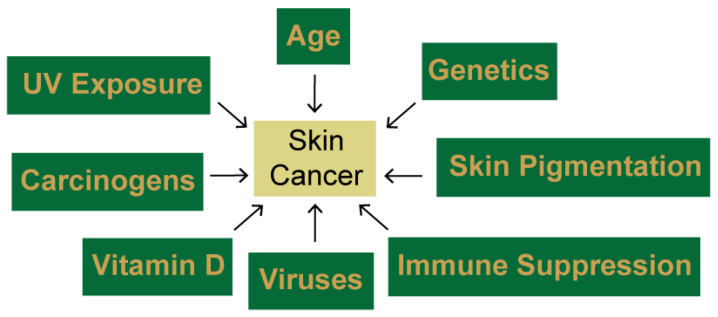
Multiple factors influence skin cancer development. Though exposure to UV radiation is a major contributor to skin carcinogenesis, other factors, including age, genetics, skin pigmentation, immunosuppression, viruses, and other carcinogens are also known to influence the likelihood of developing skin cancer.

There are several other risk factors that influence skin cancer risk, which are summarized in Figure 1. For example, most NMSCs occur in individuals over the age of 60 [14], so age is a second major risk factor. The amount of the skin pigment melanin in the skin is inversely correlated with skin cancer risk because melanin absorbs and scatters energy from UV light, thereby protecting the epidermal cells from damage. Thus, the lack of skin pigmentation commonly found in Caucasians with Fitzpatrick Type I–II skin [15], is another well-known skin cancer risk factor [16]. Recent work has shown that melanin, particularly the form known as pheomelanin, found in lightly pigmented individuals, can contribute to UV photoproduct formation after UV exposure [17]. Thus, the link between skin pigmentation and skin cancer risk is complex. Other factors that influence the likelihood of developing skin cancer are family history and genetics. Persons with xeroderma pigmentosum (XP), a genetic disorder characterized by defects in repairing UV photoproducts, have a greater than 1000-fold increased risk of developing skin cancers [18]. The immune system plays an important role in limiting skin carcinogenesis, and UV radiation is known to directly suppress immune function [19,20]. Moreover, organ transplant recipients have a significantly elevated risk of developing skin cancer because of the post-operative immunosuppressive drugs that are needed [19,20,21,22,23]. In addition to contributing to immune function, Vitamin D, which is synthesized in the skin in a UVB-dependent manner, also influences skin carcinogenesis due to its effects on DNA repair, proliferation, differentiation, and neuroendocrine pathways [24,25,26,27,28]. Lastly, exposure to certain viruses (human papilloma virus, Merkel cell polyomavirus) and other environmental carcinogens is also thought to contribute to skin carcinogenesis in certain patients [29,30,31,32]. Thus, there are many risk factors that influence the likelihood of developing skin cancer.

Though the mechanisms by which many of these risk factors contribute to mutagenesis and carcinogenesis in the skin have been studied to a reasonable extent, a new and less explored contributor is the body’s circadian rhythm. As will be discussed in greater detail below, research over the past few years has demonstrated that each of these factors is affected in some way by either the time of day or by genetic components that make up the body’s circadian clock machinery.

## 2. The Circadian Clock

### 2.1. Central and Peripheral Clocks

Though circadian (Latin for “about a day”) behaviors have been recognized and observed in organisms for centuries, it was not until the late 1970s that a genetic basis for circadian rhythms was found. Thus, as is true for understanding many aspects of biochemistry and physiology, model organisms were key to elucidating the molecular mechanism of the circadian clock. The isolation of a Drosophlia mutant with altered circadian behavior (known as period) in the 1980s ultimately led to the cloning and characterization of both the period gene and other circadian clock components. This discovery led to Jeffrey C. Hall, Michael Rosbash, and Michael W. Young being awarded the 2017 Nobel Prize in Physiology or Medicine [33]. In the 1990s, the development of the first knockout mice lacking evolutionarily conserved clock genes [34] initiated a field of research that continues today, revealing a seemingly unending number of systems and pathways that are controlled in part by the body’s circadian clock. Moreover, as will be described in greater detail below, this regulation also extends to the skin.

**Figure 2:**
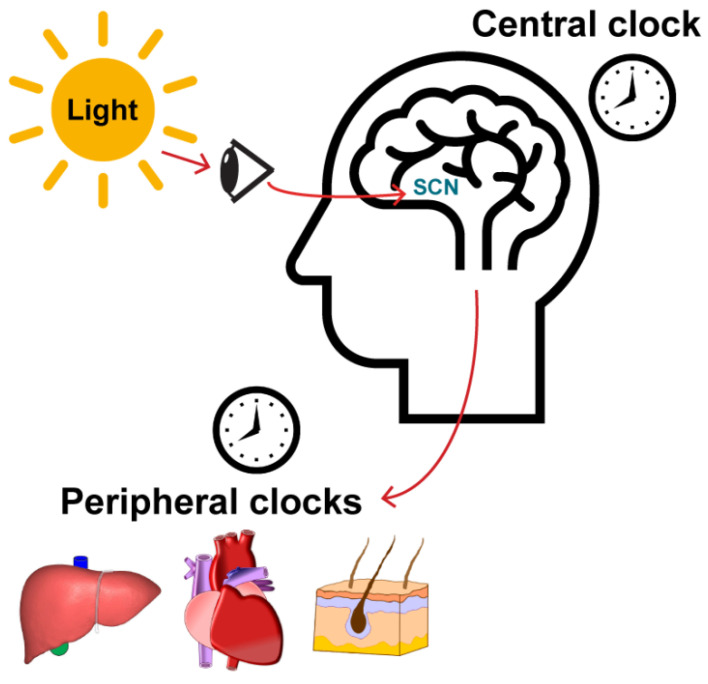
Central and peripheral circadian clocks. The body’s central or “master” clock is found in the suprachiasmatic nucleus (SCN) in the brain, which receives input from photosensitive, melanopsin-containing retinal ganglion cells via the retinohypothalamic tract. Through neuronal and hormonal signaling, the SCN then sends signals to peripheral organs to synchronize these peripheral clocks with the master clock in the brain.

A simplified model of how the body’s circadian clock is set and synchronized is provided in Figure 2. The “master clock” is found in the suprachiasmatic nucleus (SCN) in the anterior part of the hypothalamus of the brain [35]. Specialized photosensitive retinal ganglion cells in the eye sense light via the photopigment melanopsin and then this information to the SCN through the retinohypothalamic tract. The light that is sensed by the retina, therefore, allows for entrainment, or synchronization, of daily rhythms to the 24 h light-dark cycle which occurs because of the earth’s rotation. Then, via neuronal and hormonal signaling, the SCN sends signals to other peripheral organs to keep tissues synchronized to the master clock in the brain. Ultimately, these processes affect aspects of physiology that display circadian rhythmicity in humans, including blood pressure, body temperature, and the release of the hormones cortisol and melatonin.

### 2.2. Molecular Architecture of the Clock

**Figure 3:**
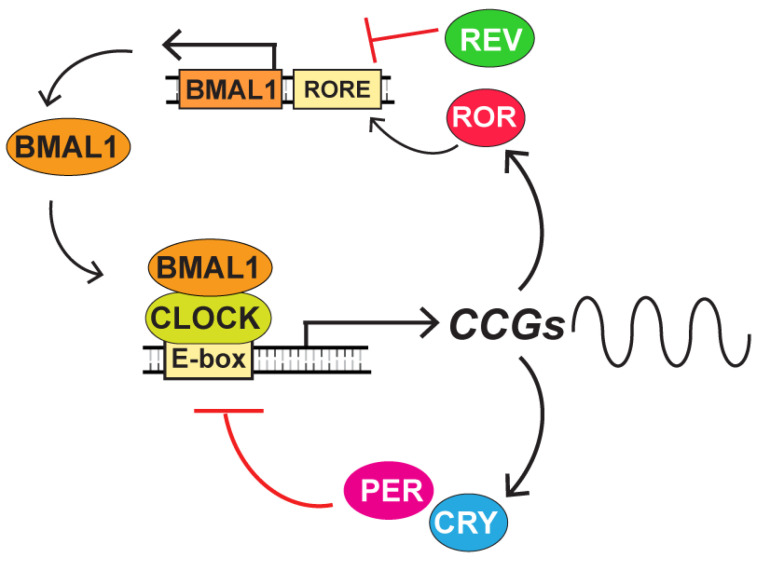
Molecular architecture of the circadian clock transcription machinery. The CLOCK-BMAL1 complex binds to E-box elements in the promoter region of clock-control genes (CCGs). These CCGs include the period (PER) and cryptochrome (CRY) gene products that feed back to inhibit CLOCK-BMAL1 activity. A secondary loop encompassing the retinoic acid-related orphan receptor (ROR) and REV–ERB gene products bind competitively to retinoic acid-related orphan receptor response elements (ROREs) in the BMAL1 promoter to regulate BMAL1 expression.

However, even in the absence of light, both the SCN and peripheral tissues can maintain biochemical and physiological rhythms that display periodicity of approximately 24 hours. Thus, the clock can function autonomously at the cellular level. At the molecular level, the circadian clock is composed of two transcription–translation feedback loops (TTFLs) [36]. A simplified model of the circadian TTFL is shown in Figure 3. The CLOCK-BMAL1 (brain and muscle Arnt-like protein-1) heterodimeric protein complex recognizes specific DNA sequences known as E-boxes in the promoter regions of genes located throughout the genome. CLOCK-BMAL1 functions as a transcription factor to control the transcription of clock-control genes (CCGs), including the period (PER) and cryptochrome (CRY) genes. Once translated into a protein and localized to the nucleus, PER–CRY complexes can bind and inhibit CLOCK-BMAL1 activity, thereby completing the negative arm of the TTFL and leading to a loss of transcriptional output. The PER and CRY proteins are eventually degraded, which allows the CLOCK-BMAL1 to promote transcription again, thus resetting the clock. In addition to this core TTFL, CLOCK-BMAL1 also activates the transcription of the retinoic acid-related orphan nuclear receptors REV-ERB and ROR. REV–ERB and ROR competitively bind to retinoic acid-related orphan receptor response elements (ROREs) found in the BMAL1 promoter either to negate or promote transcription, respectively. Thus, via controlling the abundance of BMAL1, this secondary loop also influences circadian rhythm. In addition to these core proteins, various cell signaling pathways and post-translational modifications [37] have been found to impinge upon the clock and its function. Thus, the clock is under many levels of regulation, which allows for various inputs like light, feeding, and temperature to modulate clock activity.

### 2.3. Shiftwork and Clock Disruption

With the genetic basis of circadian rhythms well-established, understanding how genetic and environmental disruptions of the clock affects physiology and disease risk becomes an important issue that remains to be fully explored. Though a number of circadian rhythm disorders exist, including various sleep–wake rhythm disorders, the molecular basis for these pathologies is largely unknown. However, mutations in PER2 are responsible for familial advanced sleep phase syndrome [38]. Humans that travel across time zones or that work night shifts may also exhibit symptoms associated with clock disruption.

About 15–20% of employees in Europe and in the U.S. are engaged in shift work that involves night work. Some experimental and observational data indicate that this type of work might lead to circadian disruption at the cellular and hormonal level. Hormonally, circadian disruptions can interfere with essential processes such as the regulation of metabolism, sleep growth factors, and other diurnal-cycle behavioral and physiological process adaptations [39,40]. Disruption to the synthesis of melatonin reduces the body’s anticarcinogenic and antioxidative defenses. At the cellular level, circadian rhythms are in partly responsible for regulating cell synthesis, mitotic mechanisms, DNA repair, and elements of the apoptotic cascade [40,41]. Given that almost 50% of the transcriptome is believed to be under circadian control [42], it is perhaps not too surprising that disruptions to the clock will affect cell function and ultimately disease pathology.

## 3. The Skin Circadian Clock

Though early analyses of circadian biology in animals focused on internal organs, such as the liver and heart, the skin has also been found to be under circadian control. Below, we review studies that addressed the molecular aspects of the clock in the skin before discussing the factors concerning clock function in the skin and the physiological processes that have been shown to be under circadian control.

### 3.1. Identifying and Characterizing the Molecular Clock in Human Skin

The first report that human skin may be governed by the genetic components of the circadian clock occurred in 2000, when the expression of CLOCK and PER1 were first demonstrated at the mRNA and protein level in cultured keratinocytes, melanocytes, and fibroblasts [43]. The widely used HaCaT keratinocyte cell line was later shown in vitro to have a functional clock [44]. Later studies showed that primary cultures of such cells could be synchronized with the glucocorticoid dexamethasone to monitor changes in clock gene expression over a couple of days [45]. Bjarnason and colleagues were the first to examine clock gene expression in tissue biopsies from the oral mucosa and skin from healthy adult males [46] and showed that the expression of Per1, Cry1, and Bmal1 at the mRNA level oscillated and peaked in early morning, late afternoon, and night, respectively. With the goal of characterizing inter-individual differences in circadian rhythmicity better, Brown et al. transduced fibroblasts isolated from skin biopsies from different donors with a lentiviral vector expressing a Bmal1 promoter and luciferase construct to monitor oscillations in circadian bioluminescence from fibroblasts [47]. The authors found widely variant circadian periods among different cell lines, suggesting that though the core clock machinery may be expressed in skin cells, its function likely varies due to additional genetic factors.

Whereas previous studies examined the expression of only a small number of genes, Spörl et al. were the first to take a more unbiased look at global gene expression throughout the day [48]. Using epidermal tissue obtained from suction blisters generated at three different times of the day (9:30 a.m., 2:30 p.m., and 7:30 p.m.), the authors carried out whole-genome microarray analyses of gene expression and observed hundreds of transcripts that displayed rhythmic expression. In addition to providing further support for the idea that the epidermis is under circadian control, their approach enabled the researchers to identify a transcription factor (Krüppel-like factor 9, or Klf9) that regulates the expression of several circadian output genes and is, itself, expressed in a rhythmic manner.

Understanding circadian rhythms in skin and other organs is made difficult by the invasive processes necessary to obtain tissue for sampling. Using hair follicle cells that remain attached to hairs plucked from either the head or chin, Akashi and colleagues demonstrated that the circadian phase of clock gene expression could be readily and accurately measured by RT-qPCR [49]. Moreover, by monitoring individual activity by a wristwatch-type device, the authors were able to correlate changes in clock gene expression with behaviors such as waking, eating, and sleeping at 3 h intervals throughout the course of a day. A similar approach using beard follicle cells supported the observation that hair follicles can be used to monitor the expression of clock gene expression at the mRNA level [50]. One study, using pubic hair follicles from nurses working either day or night shifts, found that Per2 exhibited partially reduced expression in the morning relative to daytime working nurses [51]. An additional study examining Per3 and Nr1d2 expression in beard follicle cells from a small number of individuals working either a one-night shift or continuous night shifts further revealed altered gene expression [52], though significant variation between individuals was observed.

In addition to altered circadian rhythms caused by shift work, the relation between clock gene expression in the skin and disease states was examined. Taking advantage of a Per2-luciferace viral construct and ex vivo culture of human whole hair root tissue, Yamaguchi et al. demonstrated that the circadian period length could be readily monitored in this tissue system and that older individuals with severe dementia retained clock oscillation in a manner similar to those of young and healthy subjects even though the dementia patients showed abnormal circadian behavior [53]. Altered circadian rhythms may also contribute to other diseases, such as cancer; therefore, an important issue in the cancer biology field is whether clock disruption influences carcinogenesis and whether tumors display altered clocks. Using skin biopsies of malignant melanoma and nonmalignant naevus tumors, Lengyel et al. reported that the expression of several Per and Cry genes was reduced by 30–60% relative to normal adjacent skin, whereas Clock was upregulated in nontumorous cells of melanoma biopsies [54].

Because of its abundance and ease of access, the skin has also been explored as a potential source of circadian biomarkers that could inform clinical decision making. Using the epidermis from skin punch biopsies obtained from human subjects sampled every 6 h across a 24 h period, the Hogenesch lab identified and characterized genes that displayed circadian rhythmicity [55]. The authors also compared these genes to those in mice to better identify evolutionary conservation. Then, using bioinformatics approaches and additional skin samples from a larger population of 219 individuals at a single time point, the authors were able to identify a set 29 genes that could be used to determine a circadian phase to within 3 h. More recent work from the same group took advantage of additional gene expression datasets [48,56] and reported that the clock was more robust in the epidermis than the dermis regardless of body site, age, or gender [57]. This work further refined a 12-gene expression signature that reports molecular clock phase and developed an app (SkinPhaser) to test biomarker performance in new datasets. Ultimately, this tool could be used to optimize drug timing in the emerging field of circadian medicine [58].

### 3.2. Regulators of Circadian Clock Function in the Skin

**Figure 4:**
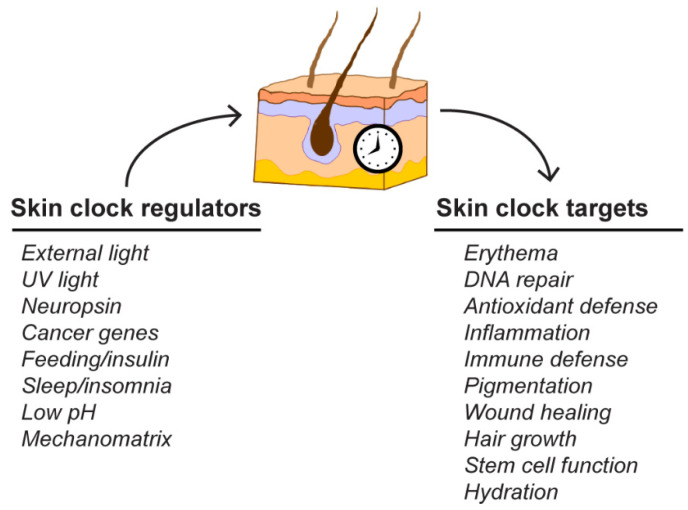
Regulators and targets of the circadian clock in the skin. Multiple factors influence circadian rhythmicity in the skin. The clock subsequently regulates many aspects of skin physiology.

Though the genetic disruption of circadian clock genes in mice proved that the clock affects various aspects of skin physiology, several studies found that additional factors can influence skin clock behavior (Figure 4). Interestingly, a recent study found that even in the absence of BMAL, skin and other tissues exhibited 24 h oscillations of the transcriptome and proteome over a few days in the absence of light, temperature, or other exogenous drivers [59]. Thus, even in the absence of a key clock gene, there may be other mechanisms that can be used to drive circadian rhythmicity.

Light is considered to the be major signal that entrains the body’s circadian clock. Though neuroendocrine mechanisms are thought to be responsible for this regulation, the mechanisms by which this takes place in peripheral tissues like the skin remain to be fully defined. Animal studies have found that light stimulation rapidly activates hair follicle stem cells via M1-type photosensitive retinal ganglion cells that signal to the SCN via melanopsin [60]. Efferent sympathetic nerves are then activated to release norepinephrine in the skin, which promotes hedgehog signaling to activate hair follicle stem cells. Whether skin cells and peripheral tissues possess their own capacity to sense light and regulate the clock remains controversial [61]. However, a recent study identified a population of melanocyte precursor cells in hair follicles that express the photopigment neuropsin (OPN5) and found that OPN5 influenced the entrainment of skin organ cultures of mouse skin exposure to violet light ex vivo [62]. In a study using human subjects, exposure to UVB wavelengths also found to alter the expression of known clock genes CRY1, CRY2, and CIART in epidermal, dermal, and even subcutaneous adipose tissue [63]. The mechanism for this response was not examined but may involve DNA damage caused by UVB radiation. Thus, various wavelengths of light are expected to have an impact on clock function in the skin by a variety of mechanisms.

Interestingly, as altered sleep schedules are known to affect various aspects of circadian physiology [64], a study in which dermal fibroblasts were isolated from the skin of idiopathic hypersomnia (IH) patients and cultured in vitro revealed dampened expression of BMAL1, PER1, and PER2 [65]. Furthermore, a BMAL1 promoter-containing luciferase reporter was used in primary fibroblasts from IH patients to show that the cells displayed a prolonged circadian period length [66].

Food is another factor affecting various organ clocks. A recent study using mice showed that time-restricted feeding (RF) shifted the circadian phase and changed the expression of about 10% of the skin transcriptome [67]. Moreover, the RF even influenced both the amount photoproducts that form in DNA after UV exposure and the expression of the nucleotide excision repair (NER) gene *XPA*. The mechanisms responsible for the effects of RF remain to be determined. However, the pancreatic hormone insulin, which is rapidly secreted in response to feeding, was shown to affect clock gene expression and circadian phase in hair follicles cultured ex vivo [68]. Thus, feeding-induced insulin release may be involved in resetting the clock in skin and other peripheral clocks.

Studies have further suggested that the process of tumorigenesis may be related to the circadian clock in the skin. For example, the overexpression of the oncogene Ras was found to disrupt the clock and increase circadian length [69]. Disruption of PTEN was similarly found to cause constitutive activation of BMAL1 in hair follicle stem cells [70]. Moreover, the presence of a tumor in the skin may also affect the skin circadian clock, as was shown when melanoma cells were injected into mouse skin [71].

### 3.3. Physiological Targets of the Skin Clock

Over the past few years, there has been a dramatic increase in our understanding of the physiological processes in the skin that are under circadian control. Summarized in Figure 4, these processes range from stem cell function and wound healing to immune defense and responses to environmental exposure. For example, cell cycle progression [72], keratinocyte proliferation [48], and stem cell function [73,74] have all been reported to show rhythmicity that likely influences reported circadian differences in processes, such as wound healing, through a variety of mechanisms [75,76,77,78]. Similarly, the clock has even been reported to affect hair follicles [79], such that hair growth is reported to occur faster in the morning [80] and be disrupted by the loss of core circadian genes [81,82]. Other fundamental properties of the skin affected include hydration [83] and pigmentation [84]. A major function of the skin is to provide a barrier against infection. Interestingly, genes encoding antimicrobial peptides [85], susceptibility to infection by herpes simplex virus 2 (HSV-2) [86], and induction of interferon-sensitive genes (ISGs) important in immune responses [87] have all been shown to be under circadian control.

Ultimately, altered circadian rhythms likely increase skin disease susceptibility. For example, experimental studies in mice have shown that genetic disruption of the circadian protein CLOCK promotes dermatitis [88]. Altering light-dark cycles similarly resulted in more pronounced effects in the Nc/Nga human atopic dermatitis mouse model [89]. Ionizing radiation-induced dermatitis, which commonly occurs in patients undergoing radiation therapy for cancer, is also stronger when the clock is disrupted by either an environmental disruption that mimics rotating shiftwork or by genetic disruption of Per1/2 [90]. Clock disruption leads to other skin conditions, such as psoriasis and time of treatment by the Toll-like receptor 7 ligand imiquimod [87,91]. Lastly, contact hypersensitivity is also negatively affected by the loss of the Clock gene [92].

## 4. Interplay between the Skin Circadian Clock, DNA Damage Responses, and Carcinogenesis

### 4.1. Induction of DNA Damage as Function of the Time of the Day

Particularly relevant to skin carcinogenesis is exposure to UVA and UVB wavelengths of sunlight, which induce different types of damage in DNA, including cyclobutane pyrimidine dimers (CPDs), pyrimidine (6-4) pyrimidone photoproducts [(6-4)PPs], and oxidative lesions such as 8-oxoguanine [93,94,95]. These lesions are potentially mutagenic if not efficiently removed by the appropriate DNA repair processes. Interestingly, one study reported that both (6-4)PPs and CPDs were induced to a slightly greater extent in mouse skin exposed to UVB radiation at night (ZT20, 2 a.m.) than in the afternoon (ZT8, 2 p.m.) [96]. This time-of-day-dependent variation in photoproduct formation was correlated with a higher proportion of epidermal cells in S phase and with the phosphorylation of the histone variant H2AX, a common marker of genomic stress. Thus, DNA may be more susceptible to UVB photoproduct formation when cells are in the process of replicating DNA. Moreover, in Bmal1 knockout mice that lack circadian clock functions, this variation in UVB photoproduct formation, proliferation, and H2AX phosphorylation was abolished. Though direct oxidative DNA adducts were not measured in this study, the authors did find that ROS levels were higher at ZT8 than at ZT20 and that this difference was correlated with gene expression involved in metabolism and cell cycle. The antiphasic nature of ROS production and DNA replication suggested that metabolic processes such as oxidative phosphorylation may be preferentially restricted in mouse skin to the times of day when DNA is not being replicated, which would help limit the likelihood of oxidative damage to DNA and potential mutagenesis. Additional work has further shown that CPD induction and oxidative metabolism in the skin are also influenced by feeding schedules [60], such that restricting it to the early or middle part of the day shifts the increased sensitivity of skin DNA to CPD formation from ZT21 (night) to ZT09 (day).

### 4.2. Circadian Regulation of DNA Repair, DNA Synthesis, and the DNA Damage Response

**Figure 5:**
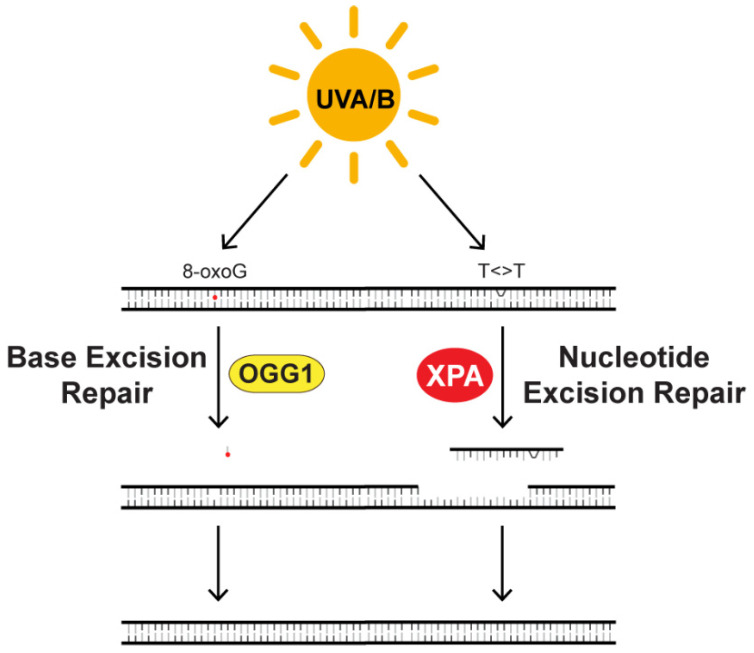
UV radiation causes the formation of lesions in DNA that are targeted for removal by DNA repair. UVA and UVB wavelengths of sunlight induce the formation of oxidative lesions in DNA, such as 8-oxoguanine (8-oxoG). These small-base lesions are targeted for removal by base excision repair (BER). UV radiation also induces the formation of photoproducts between adjacent pyrimidines in DNA (T<>T) that can only be removed by the nucleotide excision repair (NER) system. Research has shown that the expression or activity of both the BER protein OGG1 and (8-oxoguanine DNA glycosylase) and NER protein XPA (xeroderma pigmentosum group A) are controlled by the circadian clock in various organ systems.

To prevent mutagenesis, the 8-oxoguanine residues, CPDs, and (6-4)PPs induced by UV wavelengths of sunlight must be removed from the genome by specific DNA repair systems (Figure 5). The base excision repair (BER) machinery targets small-base lesions for removal, and in the case of 8-oxoguanine is initially acted upon by the enzyme OGG1 (8-oxoguanine DNA glycosylase) [97]. In a study of human subjects in which blood was collected at different times of the day, OGG1 mRNA expression and enzymatic activity were shown to oscillate in lymphocytes with peak expression/activity at 8 a.m. and low expression/activity at 8 p.m. [98]. Whether OGG1 expression exhibits a circadian pattern of expression in the skin is not yet known.

In contrast to the repair of oxidative DNA lesions, much more is known about how the circadian clock controls the repair of UV-induced CPDs and (6-4)PPs by NER. The NER system is the sole mechanism for removing UV photoproduct damage from DNA [99], and genetic disruption of this repair pathway leads to the photosensitive disorder xeroderma pigmentosum (XP) [18]. Interestingly, several lines of research have shown that NER is controlled by the circadian clock through regulation of the rate-limiting repair factor xeroderma pigmentosum group A (XPA). Early work using extracts prepared from mouse brain at different times of the day and an in vitro excision repair assay showed that NER activity oscillated in mouse brain over the course of the day and was correlated with the expression level of XPA [100]. Subsequent work in mouse skin found a similar pattern of XPA expression with high levels of XPA in the late afternoon and evening (ZT10; 5 p.m.) and low levels in the early morning (ZT22; 5 a.m.) [101]. The time-of-dependent difference in XPA expression was found to be correlated with the rate of removal of CPDs and (6-4)PPs from the skin and inversely correlated with the amount of DNA synthesis taking place in the epidermis.

**Figure 6:**
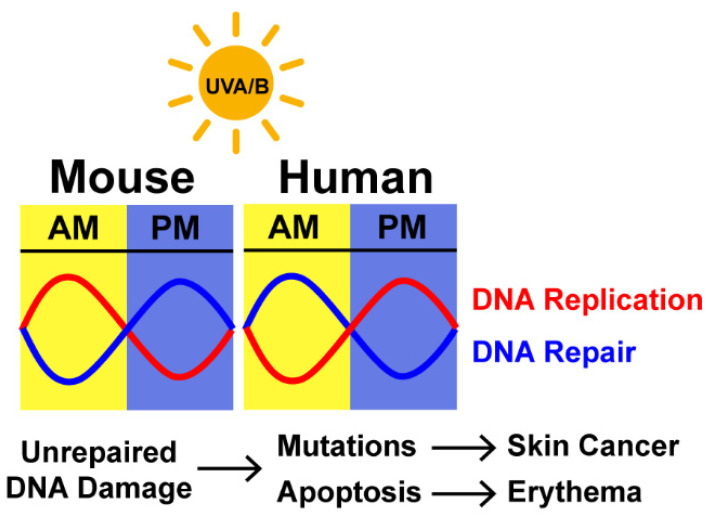
DNA replication and nucleotide excision repair in the skin display circadian rhythmicity. Studies in mice showed that XPA expression, UV photoproduct removal by NER, and DNA synthesis display circadian rhythmicity. Thus, XPA protein levels and NER are low in the early morning hours (AM) when DNA replication is high. In the afternoon and evening (PM), XPA expression and NER are high and rates of DNA replication are low. Thus, unrepaired UV photoproducts are more problematic in the early morning hours and lead to increased mutagenesis, carcinogenesis, apoptosis, and erythema. The phases of these rhythmic processes are expected to display the opposite phase in humans. Indeed, DNA synthesis has been shown to display circadian rhythmicity in human skin epidermis, such that DNA replication peaks in the mid-afternoon.

DNA adducts may be particularly problematic for mutagenesis and chromosomal stability if cells are in the process of copying the genome during the synthsis phase of the cell cycle [102,103,104]. Many studies have examined when DNA synthesis preferentially occurs in mouse and human skin [105]. In mouse skin epidermis, DNA synthesis takes place primarily during the evening and night hours [96,101,105] (Figure 6). Because NER is less efficient during this time of the day, the unrepaired adducts may lead to replication stress. Indeed, one study of mouse skin showed that markers for replication stress and the DNA damage response, including phosphorylation of the checkpoint kinase Chk1 and stabilization of the tumor suppressor p53, were elevated to a greater extent after UVB exposure in the early morning/night hours than in the late afternoon [106]. Studies of DNA synthesis in human skin as a function of the time of day showed that it appeared to display greater variability and less robust amplitudes over the circadian cycle than rodent skin [105]. This may have been due to greater inter-individual variability in human genetic and environmental factors. Nonetheless, DNA synthesis in human skin appears to show the opposite phase compared to mouse skin, such that there is greater DNA synthesis in the afternoon/evening hours than in the morning (Figure 6).

### 4.3. Circadian Regulation of UV Erythema and Skin Carcinogenesis

A common, acute consequence of excessive exposure to UV light and sunlight is erythema, or sunburn. Consistent with a role for DNA repair in limiting this outcome, work in mice showed that morning exposure to UVB induced more erythema than evening exposure [106]. Moreover, correlated with this elevated erythema, several inflammatory cytokines were found to be present at higher levels following morning UVB exposure.

Because mice are nocturnal and humans are generally diurnal, it is expected that XPA expression and UVB responses would exhibit opposite patterns of expression and function. Two studies with human subjects were carried out to address this issue. One study exposed buttock skin of human volunteers in either the morning or evening with increasing fluences of UVB light with a narrow band UVB light source and then examined skin reddening [107]. Consistent with the prediction that humans would behave differently than mice, this work showed a higher level of erythema in the skin exposed to UVB in the evening than in the morning. In contrast, another study with human subjects that used a solar simulating light source found more erythema in the morning than in the evening [108]. The reason that these two studies came to different conclusions is not clear but could be due to differences in the light sources used. The amount of 8-oxoguanines, CPDs, and (6-4)PPs induced in DNA is known to be affected by the specific wavelengths of UV exposure [93], so it is likely that that amount and type of DNA adducts induced in these two studies were different. Thus, additional work will be needed to quantify adduct formation at different times of the day and correlate this with functional consequences.

The DNA adducts induced by UVB exposure are known to generate characteristic mutations found in skin tumors [9]. Interestingly, research using mice showed that the timing of UVB exposure affects skin cancer development. Thus, SKH-1 hairless mice chronically exposed to UVB radiation in the morning (when XPA levels are low) developed tumors sooner than mice exposed in the evening [101]. Moreover, the tumors were more abundant in number and larger in diameter in the morning-exposed group in comparison to the evening-exposed group, and histopathological examination revealed the tumors to be more invasive. Thus, the combination of reduced XPA expression and increased DNA synthesis in mouse skin in the morning likely contributed to the mutagenesis that drove UVB skin carcinogenesis (Figure 6).

### 4.4. Circadian Regulation of Radiation-Induced Dermatitis

In addition to UV radiation-induced erythema and skin cancers, ionizing radiation (IR) is routinely used to treat internal solid tumors. However, many patients exhibit dermatitis at the site of radiation therapy [109], which is characterized by pain, redness, itchiness and lesions. Interestingly, a recent study showed that hairless mice lacking the Per1/2 expression or were put on a rotating light exposure schedule showed increased evidence of dermatitis following IR treatment [90]. Moreover, blood cells isolated from the mice displayed evidence of increased DNA strand breaks. Thus, because IR is known to induce oxidative lesions and direct single- and double-strand DNA breaks, it is likely that circadian clock control of DNA repair processes is relevant for ionizing radiation exposure. Though early analyses of circadian biology in animals focused on internal organs, such as the liver and heart, the skin has also been found to be under circadian control. Below, we review studies that addressed the molecular aspects of the clock in the skin before discussing the factors that influence clock function and the physiological processes that have been shown to be under circadian control.

## 5. The Circadian Clock and Disease Treatment

### 5.1. Chronopharmacology and Chronotherapy

Over the years, the circadian regulation of drug metabolism and processing has been employed in the treatment of a range of disease states, including diabetes, hypertension, peptic ulcers, and allergic rhinitis [58]. There is also interest in using chronotherapeutic approaches for skin disease, including psoriasis [110] and atopic dermatitis [111]. Although time-dictated drug administration had been demonstrated many decades ago, its application in cancer treatment was limited due to insufficient mechanistic data supporting experimental results and inconsistency among clinical trials. However, the timed administration of anti-cancer drugs is rapidly gaining attention as studies with animal and human models unveil molecular intricacies in the circadian control of biological pathways. In this regard, striking a balance between maximizing tumor responsiveness and minimizing side effects is crucial to achieving positive patient outcomes. Thus, more work is needed to understand how optimizing the timing of drug treatment can improve the treatment of skin diseases.

### 5.2. Pharmacological Modulation of the Skin Circadian Clock

**Figure 7:**
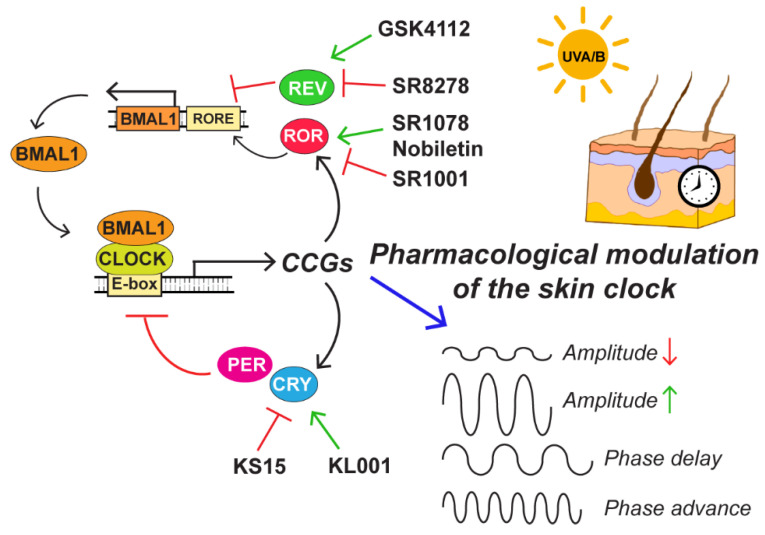
Pharmacological modulation of the skin circadian clock. Several small-molecule compounds that target circadian clock proteins have been discovered in the past few years. The application of these compounds onto human skin could potentially be used to transiently alter the amplitude or phase of CCG expression to prevent or treat skin diseases.

The realization that the circadian clock machinery affects virtually all aspects of physiology, disease pathogenesis, and treatment has led to interest in manipulating the circadian clock with small-molecule compounds [112,113,114]. Indeed, several compounds have been discovered or developed over the past few years that target specific components of the clock machinery (Figure 7), and animal studies have begun to show therapeutic benefits in metabolic disorders.

There have been a few studies that have explored the use of these compounds in treating various skin conditions. For example, the ROR agonist and flavonoid Nobiletin have been shown to prevent hyperplasia and inflammatory gene expression in UVB-exposed mouse skin [115] and inhibit skin tumors induced by the chemical carcinogen DMBA [116]. Additional studies showed that Nobiletin reduces psoriasis-like skin lesions in mice [117], mitigates oxidative stress, and enhance blood flow in a skin-reconstruct rat model [118]. Another study demonstrated that topical application of the ROR inverse agonist SR1001 on mouse skin was able to reduce inflammation induced by irritants [119]. Finally, a recent study showed that the CRY stabilizer KL001 altered the expression of proliferation and apoptotic genes and prolonged anagen in hair follicles ex vivo [120]. These data raise the possibility that drugs that target the circadian clock machinery can be employed therapeutically for a variety of different skin diseases by altering the level or timing of expression of CCGs (Figure 7).

Other recently developed compounds that remain to be explored for use in the skin include the cryptochrome inhibitor KS15 [121,122], the REV–ERB antagonist SR8278 [123], and the ROR agonist SR1078 [124]. Given that the circadian clock regulates the expression of the NER gene XPA, it may be possible to manipulate the clock machinery in human skin pharmacologically to increase XPA expression transiently during maximum sun exposure to limit erythema and mutagenesis. There has long been interest in incorporating DNA repair enzymes into topical sunscreens to prevent skin cancers [125,126]; consequently, small-molecule circadian clock modulators potentially offer a new way to increase DNA repair efficiency by promoting the expression of XPA and other DNA damage response genes.

## 6. Summary

The increase in the prevalence of melanomas and non-melanoma skin cancers among individuals with Fitzpatrick Type I–II skin types is a growing problem. Though many factors contribute to cancer risk, exposure to UV wavelengths of sunlight is considered the major cause of mutagenesis and carcinogenesis. The mechanisms by which UV radiation induces DNA damage and the DNA repair systems that target the damage for removal are well known. However, only recently has the time of day of UV exposure been examined. Studies over the past 25 years have shed new light on the genetics and biochemistry of the circadian clock, its control of gene expression, and its relevance to skin physiology and disease. In particular, the timing of UV exposure influences photoproduct formation and repair; thus, the processes of mutagenesis and skin carcinogenesis are affected by the circadian clock machinery. Finally, the more recent development of the small molecules that target clock proteins may provide new ways to prevent and treat skin disorders, including skin cancers.
